# A comprehensive study of Ecuadorian adult patients with a mild and moderate presentation of COViD-19

**DOI:** 10.1371/journal.pone.0283535

**Published:** 2023-03-23

**Authors:** Fabricio González-Andrade, Yenddy Carrero

**Affiliations:** 1 Facultad de Ciencias de la Salud y Bienestar calle Machala y Sabanilla, Universidad Tecnológica Indoamérica, Quito, Ecuador; 2 Facultad de Ciencias Médicas, Unidad de Medicina Traslacional, Universidad Central del Ecuador, Quito, Ecuador; 3 Facultad de Ciencias de la Salud, Carrera de Medicina, Universidad Técnica de Ambato, Ingahurco, Ambato, Ecuador; Mahidol Oxford Clinical Research Unitl (MORU), THAILAND

## Abstract

**Aim:**

To characterize non-hospitalized patients with mild and moderate clinical presentation.

**Methods:**

We performed an epidemiological, observational, descriptive, and cross-sectional study carried out in Ecuador, with 1,447 participants between 18 and 66 years, non-hospitalized, with a molecular RT-PCR test for SARS-CoV2. We analyzed demographic characteristics according to sex, age group, clinical findings, behavior after diagnosis, family and social behavior, sequelae, clinical evolution, type of exposure, and personal history.

**Results:**

The sample analyzed had a mean age of 37 years (95% CI 18–66), women 713 individuals (49.27%), men 733 individuals (50.66%). Age group distribution was 18–30 years, 524 individuals (36.29%), 31–45, 538 individuals (37.26), and more of 45 years, 382 individuals (26.46%). 1416 individuals were mestizos (97.99%). According to the province of residence from Pichincha were 1019 patients (70.52%), followed by Imbabura, 93 patients (6.44%), and the others 335 (23.15%) patients come from all over the country. In women, the most common findings were fever >38°C (54.40%), sputum (27.43%) and hypoxia (16.32%); HTN (5.75%) and hypercholesterolemia (3.69%). Men were more prevalent in all other findings. Comorbidities were more prevalent in all those over 45 years of age. COVID-19 antibodies test was positive in 416 patients (28.85%). Neuropsychiatric symptoms such as sleep disorders, generalized anxiety disorder, depressed mood, and chronic fatigue were more prevalent in men than women. Still, generalized anxiety disorder and chronic fatigue were more common in individuals of 31 to 45 years. 868 patients (60.07%) were in contact with a known infected person, 318 patients (22.02%) were health workers, and 782 patients (57.63%) were informed about work exposure. 545 patients (37.72%) were overweight, primarily women 310 (42.29%). 609 patients (42.65%) showed symptoms after the acute period, and 331 individuals (23.49%) reported some sequelae.

**Conclusion:**

The epidemiological and clinical behavior of hospitalized and critical patients differs greatly from ambulatory or mild or moderate symptoms. It is essential to highlight those non-hospitalized patients constitute the predominant population of patients, hence the importance of adequate management that would directly affect the development of complicated forms and, consequently, the collapse of healthcare centers. It is vitally important to open more investigations that compare hospitalized and outpatient patients to have a clearer picture of the epidemic.

## Introduction

Although it seems that the SARS-CoV-2 pandemic has subsided, many cases persist, particularly those with mild or moderate symptoms that can be confused with other similar and milder pathologies [1]. The pandemic is still not over despite global vaccination and epidemiological control efforts. The symptoms in mild-moderate clinical presentations of COViD-19 vary from severe cases worldwide, and human behavior directly influences the clinical findings. Some studies described atypical presentations, and older adults and people with medical comorbidities may have a late manifestation of fever and respiratory symptoms. In other words, the clinical description is not specific [2]. Indeed, coronaviruses are pathogenic agents that can cause a variety of respiratory, enteric, liver, and neurological symptoms [3]. Infection by these agents, for the most part, can go unnoticed or produce clinical pictures ranging from the common cold to more acute severe pictures such as those produced by SARS and MERS-CoV [4]. In the neo-agent SARS-CoV-2, the clinical presentation varies from mild-moderate to severe cases [5]. The clinical forms of COVID-19 present as mild, moderate, or severe disease. Most patients are asymptomatic carriers who have the potential to be contagious to others they come into close contact with. Others have a mild illness similar to influenza infection that cannot be differentiated from a common upper respiratory tract infection.

In COViD-19 infection, the lung parenchyma is involved, causing a type of atypical pneumonia that hinders adequate ventilation and gas exchange, ending in ventilatory assistance, hospitalization, and ultimately death in many cases. However, the predominant clinical spectrum is characterized by mild-moderate respiratory manifestations in at least 80% of cases. The remainder is distributed in patients with a moderate presentation who merit hospital stays in 15% of cases, and severe pneumonia, in 5% of cases, which deserve management in an intensive care unit (ICU), intubation, parenteral drugs, life support, among others [6]. A clinical presentation by cough, anosmia, ageusia, pharyngeal discomfort, dyspnea of different degrees, and fever and touch to the general state characterizes it. Some research has found a lower incidence of diarrhea, abdominal pain, acute abdomen, various dermatoses, and organ failure in severe cases [7]. However, the determinants in these patients’ clinical evolution and prognosis could relate to the clinical and demographic characteristics such as age, associated comorbidities, immune competence, the therapy used, management in different healthcare centers worldwide, and health conditions of their human behavior [8]. Observations of infected patients noted that those over 65 years and males predominated [9]. However, as the pandemic progresses, selective pressure on virion infectivity has been seen, affecting young people, which suggests that other factors influence, such as immunogenicity [10].

Associated comorbidities are risk factors for the severe clinical presentation of the disease. Pathological histories such as hypertension (HTN), type 2 diabetes mellitus (T2DM), or chronic kidney disease (CKD) are linked to the severe form of COViD-19 [11]. Obesity is another variable in these patients’ slow evolution [12], a risk factor for the most common 20 chronic conditions [13]. A factor correlated with the disease’s severity is psychobiological habits such as tobacco smoking. A group of public health experts established that smokers are more likely to develop severe symptoms in the case of COViD-19 [14]. The heterogeneous behavior of the disease, according to age groups, geographical areas, and possible circulation of different strains, plays a critical role in the study of COViD-19 in Ecuador, coupled with the under-registration of cases, little knowledge of the dynamics of infection and distribution of outbreaks, as insufficient detailed data existent [15–18]. The clinical-epidemiological characteristics are still unknown, especially in symptomatic patients with mild-moderate symptoms, which leads to erroneous diagnoses and treatments [19]. Thus, it is crucial to identify the risk factors and the infection’s clinical characteristics in a well-defined population to establish essential guidelines for the containment of the viral spread. Despite the rapid advances in understanding this new respiratory syndrome, data characterizing its epidemiology, clinical features, and response to treatment are fewer in lower and middle-income countries like Ecuador [20].

This paper aims to characterize non-hospitalized patients with mild and moderate clinical presentation.

## Methods

### Research design

We performed an epidemiological, observational, and cross-sectional study.

### Settings

We performed this study in Ecuador. The study was conducted during the years 2020 and 2021. The surveys were applied throughout the country randomly, but most of the patients came from Quito, province of Pichincha. Not all patients present the same findings since there was a lot of variability in the results, mainly due to the condition being a new disease.

### Participants

Any resident in Ecuador, alive, with an infection with CoViD19, a positive RT-PCR molecular test, non-hospitalized, with mild-moderate clinical presentation. We randomly selected all patients.

### Study size

We analyzed 1447 participants between 18 and 66 years, according to sex and age groups. We collected the data of all patients with symptoms and an RT-PCR+.

### Definitions

We defined mild illness in individuals with various signs and symptoms, e.g., fever, cough, sore throat, malaise, headache, and muscle pain, without dyspnea or abnormal imaging. We consider moderate disease when individuals have lower respiratory disease, evidenced by clinical evaluation or imaging and oxygen saturation (SaO2) greater than 93% in ambient air at sea level.

### Inclusion criteria

Patients in Ecuador of all ages, both sexes, and any ethnic group, randomly selected, non-hospitalized, with a positive RT-PCR molecular test and active infection.

### Exclusion criteria

Patients hospitalized with a severe or critical clinical presentation; with post-intensive care syndrome (PICS), anyone who survives a critical illness that justifies admission to an ICU is susceptible to developing post-intensive care syndrome, characterized by the appearance or worsening of cognitive, physical or mental health of the patient after discharge. We differentiated between this syndrome caused by COVID-19 or caused by just being in the ICU. Patients with symptoms less than four weeks after discharge from the hospital or leaving isolation or who have received the flu vaccine in the last six months or pneumococcus in the previous five years.

### Variables

We analyzed demographic characteristics according to sex, age group, clinical findings, behavior after diagnosis, family and social behavior, sequelae, clinical evolution, type of exposure, and personal history.

### Data sources

We conducted direct interviews with patients maintaining safety measures, and when not possible (isolated patients), we conducted telephone interviews.

### Bias avoidance

The same person conducted the interviews using a standardized data collection form.

### Statistical methods

We analyzed data with the SPSS^©^ software version 22.0. We used descriptive and inferential statistics. Comparing the differences of variables, we used chi-squared for proportional data and t-test or ANOVA for normally distributed continuous data or their nonparametric tests for skewed data. Logistic regression was used to assess the factors for a binary endpoint. For some analyses, we stratified by age: 18 to 30 years, 30 to 45 years, 45 to 65 years, and ≥65 years, and for other analyses, we had one group > 45 years. A p-value < 0.05 was considered statistically significant.

### Exposure

Mild and moderate COVID-19 infection.

### Ethical issues

All the information obtained was filled out in the data collection sheet in a completely anonymous way. No data was filled that could reveal the patient’s identity directly or indirectly. All patients signed informed Consent. The information obtained was confidential, and all individual data was anonymous. Our research group keeps the data. All methods were carried out under relevant guidelines and regulations.

### Institutional Review Board (IRB)

We received IRB approval from the Ethics Committee on Research in Humans from Carlos Andrade Marín Hospital on July 6^th^ of, 2020, with the code IESS-HCAM-CEISH-2020-200-DF. This research is part of the project entitled “Clinical, neurological, and radiological characterization of adult Ecuadorian patients with SARS-CoV2 infection to establish risk prediction models based on the phenotype”. All patients provided the information voluntarily, signed an Informed Consent, or gave their Consent via telephone, in both cases, with the presence of a witness. The information obtained is confidential, and we anonymize all individual data. Our research group keeps the data. All methods followed the relevant Helsinki Declaration, developed by the World Medical Association, outlining the ethical standards for research on human participants. Also, we follow other guidelines and national regulations.

## Results

**Table 1** shows the distribution of non-hospitalized patients infected with COVID-19 according to sex, age group, and clinical findings. **Table 2** shows the distribution of non-hospitalized patients infected with COVID-19 according to sex, age group, behavior after diagnosis, family and social behavior, sequelae, and clinical evolution. **Table 3** shows the distribution of non-hospitalized patients infected with COVID-19 according to sex, age group, type of exposure, and personal history.

**Table 1:** Distribution of non-hospitalized patients infected with COVID-19 according to sex, age group, and clinical findings.

Clinical findings	Total	Sex (n = %)		p-value	Age group (n = %)			p-value
		Women	Men		18–30 y	31–45 y	>45 y	
**Comorbidities**								
HTN	80 (5.54)	38 (5.33)	42 (5.75)	0.730	9 (1.72)	20 (3.72)	51 (13.35)	0.001*
T2DM	31 (2.15)	19 (2.67)	12 (1.64)	0.177	3 (0.58)	7 (1.3)	21 (5.5)	0.001*
Hipercholesterolemia	43 (2.98)	16 (2.24)	27 (3.69)	0.105	5 (0.96)	21 (3.9)	17 (4.45)	0.003*
**Diagnostic behavior**								
COVID-19 antibodies test positive	416 (28.85)	217 (30.52)	199 (27.22)	0.167	151 (28.87)	173 (32.34)	92 (24.08)	0.025*
**General symptoms**								
Cefalea	970 (67.22)	483 (67.84)	487 (66.62)	0.623	339 (64.82)	361 (67.23)	269 (70.6)	0.187
Fatige	896 (62.14)	460 (64.52)	436 (59.81)	0.037*	313 (59.85)	331 (61.64)	251 (66.05)	0.157
Cough	842 (58.39)	436 (61.15)	406 (55.69)	0.036*	286 (54.68)	311. (57.91)	242 (63.68)	0.025*
Anosmia	839 (58.18)	424 (59.47)	415 (56.93)	0.328	314 (60.04)	326 (60.71)	198 (52.11)	0.019*
Myalgia and arthralgia	804 (55.76)	403 (56.52)	401 (55.01)	0.563	272 (52.01)	301. (56.05)	232 (61.05)	0.026*
Fever >38°C	737 (51.15)	341 (47.83)	396 (54.40)	0.013*	251 (47.99)	266. (49.63)	218 (57.37)	0.015*
Dyspnea	629 (43.62)	317 (44.46)	312 (42.80)	0.525	196 (37.48)	245 (45.62)	185 (48.68)	0.002*
Disgeusia	628 (43.55)	327 (45.86)	301 (41.29)	0.080	233 (44.55)	242 (45.07)	152 (40)	0.264
Diarrhea	602 (41.69)	305 (42.78)	297 (40.63)	0.408	177 (33.84)	245 (45.62)	180 (47.12)	0.000*
Nose obstruction	493 (34.14)	257 (36.04)	236 (32.28)	0.132	190 (36.33)	176 (32.77)	128 (33.51)	0.446
Sputum	374 (25.94)	174 (24.40)	200 (27.43)	0.189	113 (21.61)	143 (26.63)	118 (31.05)	0.005*
Abdominal pain	349 (24.17)	181 (25.39)	168 (22.98)	0.286	100 (19.12)	142 (26.44)	107 (28.01)	0.003*
Nausea/vomiting	335 (23.20)	193 (27.07)	142 (19.43)	0.001*	113 (21.61)	131 (24.39)	91 (23.82)	0.533
Dizziness	323 (22.43)	184 (25.88)	139 (19.07)	0.002*	109 (20.84)	127 (23.69)	88 (23.22)	0.503
Odynophagia	310 (21.47)	170 (23.84)	140 (19.15)	0.030*	103 (19.69)	111 (20.67)	96 (25.13)	0.122
Hypoxia	224 (15.53)	105 (14.73)	119 (16.32)	0.403	66 (12.62)	71 (13.22)	87 (22.89)	0.001*
Skin lesions	133 (9.21)	76 (10.66)	57 (7.80)	0.037*	53 (10.13)	44 (8.19)	36 (9.42)	0.544
Hematic lesions	38 (2.64)	25 (3.51)	13 (1.78)	0.040*	12 (2.29)	12 (2.24)	14 (3.67)	0.339
**Neuropsychiatric symptoms**								
Sleep disorder	599 (41.45)	319 (44.74)	280 (38.25)	0.012*	187 (35.69)	229. (42.57)	183 (48.03)	0.001*
Generalized Anxiety Disorder	363 (25.17)	199 (27.99)	164 (22.44)	0.015*	122 (23.28)	151 (28.07)	90 (23.81)	0.153
Depressed mood	451 (31.45)	263 (37.25)	188 (25.82)	0.001*	144 (27.64)	168. (31.52)	139 (36.77)	0.014*
Chronic fatigue	176 (12.21)	103 (14.49)	73 (10)	0.009*	51 (9.75)	77 (14.31)	48 (12.7)	0.073

Note

* Significant differences in the proportions p-value <0.05, based on the Chi-square test. Not all patients present the same findings, since there was a lot of variability in the results, especially due to the condition of being a new disease.

**Table 2:** Distribution of non-hospitalized patients infected with COVID-19 according to sex, age group, behavior after diagnosis, family and social behavior, sequelae, and clinical evolution.

		Sex (n = %)			Age group (n = %)			
	Total	Women	Men	p-value	18–30 y	31–45 y	>45 y	p-value
**Social behavior after diagnosis**								
The length of time of the illness (mean (SD))^2/^	19.51 (13.28)	19.68 (12.92)	19.35 (13.64)	0.288	18.27 (12.57)	19.12 (13.12)	21.73 (14.16)	0.001**
CI-95% days elapsed	19–22	19–22	19–22		17–19	17–20	20–23	
Total, isolation type (n (%))^1/^								
Voluntary	921 (64.41)	451 (63.7)	470 (65.1)	0.581	350 (67.57)	314 (58.8)	255 (67.82)	0.003*
Mandatory	509 (35.59)	257 (36.3)	252 (34.9)		168 (32.43)	220 (41.2)	121 (32.18)	
**Family and social behavior**								
Infected relatives number (media (DE))^2/^	2.04 (1.51)	2.11 (1.56)	1.97 (1.46)	0.166	2.20 (1.53)	1.95 (1.52)	1.98 (1.47)	0.019**
CI-95% infected relatives number	1.90–2.12	1.90–2.21	1.81–2.13		2.01–2.39	1.77–2.12	1.78–2.18	
Sequelae								
Persistent symptoms after the acute period (n (%))^1/^	609 (42.65)	338 (47.94)	271 (37.48)	0.001*	205 (39.5)	236 (44.36)	167 (44.53)	0.193
Type of persistent symptoms after the acute period (n (%))^1/^								
Fatige	70 (31.67)	38 (30.89)	32 (32.65)	0.613	19 (25.68)	24 (28.57)	14 (22.22)	0.043*
Headache	57 (25.79)	36 (29.27)	21 (21.43)		0 (0)	3 (3.57)	2 (3.17)	
Back pain	23 (10.41)	10 (8.13)	13 (13.27)		6 (8.11)	5 (5.95)	12 (19.05)	
Hyposmia	23 (10.41)	14 (11.38)	9 (9.18)		6 (8.11)	6 (7.14)	5 (7.94)	
Myalgias	17 (7.69)	10 (8.13)	7 (7.14)		2 (2.7)	1 (1.19)	5 (7.94)	
Odynophagia	14 (6.33)	6 (4.88)	8 (8.16)		7 (9.46)	3 (3.57)	4 (6.35)	
Arthralgias	8 (3.62)	3 (2.44)	5 (5.1)		20 (27.03)	34 (40.48)	17 (26.98)	
Lung pain	5 (2.26)	4 (3.25)	1 (1.02)		3 (4.05)	1 (1.19)	0 (0)	
Dysgeusia	4 (1.81)	2 (1.63)	2 (2.04)		11 (14.86)	7 (8.33)	4 (6.35)	
**Clinical evolution (n (%))** ^**1/**^								
Total, recovery	1055 (74.88)	505 (72.66)	550 (77.03)	0.130	399 (77.33)	395 (74.81)	259 (71.35)	0.255
Complications	23 (1.63)	14 (2.01)	9 (1.26)		7 (1.36)	7 (1.33)	9 (2.48)	
Sequelae	331 (23.49)	176 (25.32)	155 (21.71)		110 (21.32)	126 (23.86)	95 (26.17)	
Deceased (n (%))	3 (0.21)	2 (0.28)	1 (0.14)	0.620	1 (0.19)	0 (0.00)	2 (0.53)	0.620

Note

* Significant differences in the proportions p-value <0.05. 1/ based on the Chi-square test; 2/ based on the Mann-Whitney test. Not all patients present the same findings, since there was a lot of variability in the results, especially due to the condition of being a new disease.

**Table 3:** Distribution of non-hospitalized patients infected with COVID-19 according to sex, age group, type of exposure and personal history.

		Sex (n = %)			Age group (n = %)			
	Total	Women	Men	p-value	18–30 y	31–45 y	>45 y	p-value
Exposure type								
Contact with a known infected person	868 (60.07)	434 (60.96)	434 (59.21)	0.498	334 (63.74)	309 (57.54)	223 (58.38)	0.090
Health worker	318 (22.02)	187 (26.26)	131 (17.9)	0.001*	148 (28.3)	117 (21.79)	53 (13.87)	0.001*
Type of exposure with infected people								
Work exposure	782 (57.63)	352 (52.85)	430 (62.23)	0.001*	267 (54.16)	346 (68.51)	169 (47.47)	0.001*
School exposure	27 (1.99)	13 (1.95)	14 (2.03)		9 (1.83)	14 (2.77)	4 (1.12)	
Family exposure	413 (30.43)	240 (36.04)	173 (25.04)		169 (34.28)	103 (20.4)	138 (38.76)	
Public places exposure	135 (9.95)	61 (9.16)	74 (10.71)		48 (9.74)	42 (8.32)	45 (12.64)	
Perception of the place where it was infected								
Work	804 (57.8)	363 (52.92)	441 (62.55)	0.001*	273 (54.49)	355 (68.53)	176 (47.57)	0.001*
School	3 (0.22)	2 (0.29)	1 (0.14)		3 (0.6)	0 (0)	0 (0)	
Family	425 (30.55)	247 (36.01)	178 (25.25)		176 (35.13)	109 (21.04)	137 (37.03)	
Public places	132 (9.49)	63 (9.18)	69 (9.79)		40 (7.98)	48 (9.27)	45 (12.16)	
Friend’s visit	9 (0.65)	3 (0.44)	6 (0.85)		4 (0.8)	1 (0.19)	4 (1.08)	
Medical appointment	18 (1.29)	8 (1.17)	10 (1.42)		5 (1)	5 (0.97)	8 (2.16)	
Personal History								
BMI Normal	741 (51.28)	385 (54.07)	356 (48.57)	0.001*	333 (63.55)	235 (43.76)	173 (45.29)	
Overweight	545 (37.72)	235 (33.01)	310 (42.29)		157 (29.96)	234 (43.58)	154 (40.31)	0.001*
Mild obesity	124 (8.58)	68 (9.55)	56 (7.64)		24 (4.58)	52 (9.68)	46 (12.04)	
Moderate obesity	24 (1.66)	17 (2.39)	7 (0.95)		5 (0.95)	10 (1.86)	9 (2.36)	
Severe obesity	5 (0.35)	1 (0.14)	4 (0.55)		1 (0.19)	4 (0.74)	0 (0)	
Malnourished	6 (0.42)	6 (0.84)	0 (0)		4 (0.76)	2 (0.37)	0 (0)	

Note

* Significant differences in the proportions p-value <0.05, based on the Chi-square test. Not all patients present the same findings, since there was a lot of variability in the results, especially due to the condition of being a new disease.

The sample analyzed had a mean age of 37 years (95% CI 18–66), women 713 individuals (49.27%), men 733 individuals (50.66%). Age group distribution was 18–30 years, 524 individuals (36.29%), 31–45, 538 individuals (37.26), and more of 45 years, 382 individuals (26.46%. 1416 individuals were mestizos (97.99%). According to the province of residence from Pichincha were 1019 patients (70.52%), followed by Imbabura, 93 patients (6.44%), and the others 335 (23.15%) patients come from all over the country.

In women, the most common findings were fever >38°C (54.40%), sputum (27.43%) and hypoxia (16.32%); HTN (5.75%) and hypercholesterolemia (3.69%). Men were more prevalent in all other findings. Comorbidities were more prevalent in all those over 45 years of age. COVID-19 antibodies test was positive in 416 patients (28.85%). Neuropsychiatric symptoms such as sleep disorders, generalized anxiety disorder, depressed mood, and chronic fatigue were more prevalent in men than women. Still, generalized anxiety disorder and chronic fatigue were more common in individuals of 31 to 45 years. 868 patients (60.07%) were in contact with a known infected person, 318 patients (22.02%) were health workers, and 782 patients (57.63%) reported work exposure. 545 patients (37.72%) were overweight, primarily women 310 (42.29%). 609 patients (42.65%) showed symptoms after the acute period, and 331 individuals (23.49%) reported some sequelae.

Other interesting findings were that 99.43% (1404) of patients were in home isolation, and the remaining patients did continue working; 41 patients (2.84%) were infected by staying in a foreign country. In 988 patients (68.37%), the diagnosis was performed by a physician, and the remaining patients were diagnosed by a registered nurse, laboratory worker, or self-diagnosed. Sixteen (1.71%) were pregnant, and 123 patients (8.52%) were smokers. Three participants patients died after the interview with the infection.

## Discussion

This observational study reflects the characteristics of the non-hospitalized Ecuadorian population, with mild and moderate clinical presentations. Despite the significant challenges found in the specialized healthcare centers for patients with COVID-19, it is essential to highlight those non-hospitalized patients constitute the predominant population. Hence, properly managing these patients would directly affect the development of complicated forms and, consequently, the collapse of healthcare centers and possible reinfections. This study is of great importance since there are scarce data on the clinical and epidemiological characteristics of COVID-19 patients in Ecuador.

We found that the patients’ mean age of our recruited adults was 47 years. Although demographic changes may be more remarkable, the male sex continues to be the most affected. This predisposition is still not entirely apparent. Indeed, the male sex is mainly affected by having a higher BMI, tending toward the complication of obesity. This study obtained a considerable percentage of the infected healthcare personnel, represented by medical doctors, nurses, and health workers.

Nonetheless, healthcare workers are on the front lines in the fight against the pandemic. One risk factor for increasing the disease’s severity is the patient’s metabolic condition. The results show a small percentage of patients who presented some comorbidity, although it has been described that the most frequent was HTN. Metabolic processes are essential mediators of host defense mechanisms that protect against physiological damage during infections and enable survival.

The most frequent symptoms in this study were headache, fatigue, cough, anosmia, fever, and dyspnea. Likewise, we found significant differences concerning sex and symptoms, such as fatigue, fever >38°C, nausea/vomiting, odynophagia, skin lesions, and blood abnormalities. These findings are similar to scientific literature but with some exceptions. There are more than 50 signs and symptoms at least related directly to the infection.

The diagnostic tests focused on symptomatic patients without identifying asymptomatic or pre-symptomatic patients. These individuals play an essential role in the transmission of the disease. The daily reports on the behavior of COVID-19 issued by the Ecuadorian government do not adequately represent the growth in the number of infected each day, nor the natural behavior of the epidemic, affecting possible control measures. On the other hand, the high percentage of unexposed healthy subjects with a pre-existing immunity suggests that a part of the Ecuadorian population is likely to have SARS-CoV-2 reactive T-cells [21], which suggests that it is likely that a part of the Ecuadorian population has T cells reactive to SARS-CoV-2 without a more exact picture established. Increasing the number of clinical and epidemiological research with the molecular characterization of circulating viral variants and the immune response and certain protective factors related to genetic components in the population would be interesting.

Among the main epidemiological findings, we show that the nutritional status of non-hospitalized patients predominates the normal nutritional status, accompanied by overweight. However, this situation is the opposite and different for hospitalized patients. There is growing evidence that obesity is one of the most common conditions associated with COVID-19, and morbid obesity is significantly associated with the disease’s severe presentation. Obesity, especially abdominal obesity, accompanied by low-grade inflammation, could be modified by amplifying the exacerbated immune response to COVID-19. Obese people also frequently have other cardio-metabolic conditions that increase the risk of SARS-CoV-2 infection. Therefore, we infer that a healthy nutritional status is characteristic of the disease’s mild presentation. Indeed, the genetic condition with a tendency towards obesity represents a greater susceptibility to infection and severe presentation, which influences the presence of other comorbidities.

Social behavior is critical to establish knowledge about the diagnosis of the disease. Voluntary social isolation was the most frequent practice. Despite this, experts in the follow-up of asymptomatic patients with mild and moderate symptoms suggest that symptomatic patients should continue to maintain preventive isolation, with frequent hand washing, the social distancing of at least one meter, and the use of masks have already reduced viral transmission. As social determinants of health measure, high social risk can increase the risk of SARS-CoV-2 infection. In Ecuador, a study showed that high levels of education were related to more virus acknowledgment [22]. However, they were less assertive about the virus’s characteristics and used empirical and unproven treatments. This factor is crucial and directly influences the population’s behavior, impacting the rebound or re-emergence of cases.

One of the clinical-epidemiological characteristics of morbidity and the tendency to complications is the persistence of symptoms. We observed a range of remaining symptoms, including cough, shortness of breath, fever, sore throat, chest pain, palpitations, cognitive deficits, myalgia, neurological symptoms, rash, and diarrhea. Regarding the clinical sequelae, our study observed that about half of the non-hospitalized patients had symptoms after the infection; the most common were fatigue and headache. Many studies discuss long-term sequelae; however, this topic should be studied more deeply. Patients with mild or moderate symptoms remitted the infection in the first week. In contrast, severely ill patients cannot eliminate the virus optimally, leading to the disease’s critical form. The mechanism by which post-infectious symptoms persist remains unknown.

Maintaining strict prevention and control measures is essential—the lack of knowledge of the virus’s biology limits future prevention and vaccination campaigns. Outpatients are essential in the viral spread, considering reported reinfection cases and new circulating viral variants. Indeed, educational health and communication programs should emphasize explaining the essential molecular characteristics of SARS-CoV-2; thus, the population can adhere to the measures they must adopt, the possible complications inherent to the infection, and the control program restrictions needed in favor of collective health.

One last important issue is that tests should always be performed on patients with symptoms such as fever, fatigue, headache, malaria, and COVID-19. In the case of challenges due to the COVID-19 pandemic, a malaria diagnosis should be considered for all fever cases in endemic countries. On the other hand, patients with COVID-19-related symptoms that are negative for malaria must undergo isolation to exclude COVID-19 until the repetition of the virological sample, thus reducing the potential risk of transmission.

This study has some limitations. Few studies talk about confirmed non-hospitalized infected patients with mild-moderate illnesses. Due to the pandemic’s size, we believe the sample size could be more extensive. Furthermore, many asymptomatic patients refuse to recognize the disease or confuse the symptoms, making it challenging to identify potential patients. Additionally, the availability and access to molecular diagnostic tests are still reduced in Ecuador. The future perspectives of this research will be the clinical follow-up of these patients and the follow-up with computed tomography and respiratory function tests. It is possible to generalize this research with a prospective design that includes a larger sample.

## Conclusion

The epidemiological and clinical characteristics of hospitalized and critical patients differ greatly from ambulatory patients or those with mild or moderate symptoms. It is essential to emphasize that non-hospitalized patients constitute the predominant patient population, hence the importance of adequate management that would directly impact the development of complicated forms and, consequently, the collapse of healthcare centers and possible infections. It is essential to carry out more research that includes hospitalized and ambulatory patients to have a clearer picture of the epidemic in the country, which will allow more specific and forceful control measures to be taken to reduce cases and deaths due to COVID-19.

## Supporting information

S1 Data (XLSX)

## Abbreviations

CKD: chronic kidney disease
COVID-19: Coronavirus disease 2019
HTN: hypertension
ICU: intensive care unit
MERS-CoV: Middle East respiratory syndrome coronavirus
OR: Odds ratio
PCR+ test: polymerase chain reaction test positive
PICS: post-intensive care syndrome
RT-PCR: reverse transcription polymerase chain reaction
SaO2: oxygen saturation
SARS-CoV-2: severe acute respiratory syndrome coronavirus
T2DM: type 2 diabetes mellitus
